# Metal-Based Nanoparticles for the Treatment of Infectious Diseases

**DOI:** 10.3390/molecules22081370

**Published:** 2017-08-18

**Authors:** Blessing Atim Aderibigbe

**Affiliations:** Department of Chemistry, University of Fort Hare, Alice Campus, Eastern Cape 5700, South Africa; blessingaderibigbe@gmail.com; Tel.: +27-040-602-2266

**Keywords:** infectious diseases, metal-based therapeutics, influenza, HIV, herpes, malaria, cervical cancer, tuberculosis

## Abstract

Infectious diseases can be transmitted and they cause a significant burden on public health globally. They are the greatest world killers and it is estimated that they are responsible for the demise of over 17 million people annually. The impact of these diseases is greater in the developing countries. People with compromised immune systems and children are the most affected. Infectious diseases may be caused by bacteria, viruses, and protozoa. The treatment of infectious diseases is hampered by simultaneous resistance to multiple drugs, indicating that there is a serious and pressing need to develop new therapeutics that can overcome drug resistance. This review will focus on the recent reports of metal-based nanoparticles that are potential therapeutics for the treatment of infectious diseases and their biological efficacy (in vitro and in vivo).

## 1. Introduction

Infectious diseases are responsible for many deaths worldwide and are caused by fungi, viruses, bacteria, and parasites. Infectious diseases can be classified as emerging and re-emerging infectious diseases. The diseases which are new are referred to as emerging infectious diseases, whereas re-emerging infectious diseases refer to infections which are not new, but suffer from drug-resistance when they reappear, thereby making them difficult to treat or control [1,2,3]. Although the human immune system has the ability to defend the body from infections, some infections however are easily transmitted, while others are very contagious and virulent in nature [4]. On the other hand, some of infections are not contagious. Overall, infections can be transmitted when microorganisms causing infections enter the host body via natural orifices resulting in the microorganism growing at the site of entry followed by multiplication in the host cells resulting in tissue damage [4]. However, it is important to mention that some microorganisms can replicate in the extracellular spaces within the body resulting in tissue damage.

The treatment of infectious diseases is hampered by drug resistance, indicating that there is a serious need to develop new therapeutics which can overcome drug resistance. Therapeutic agents such as antibody-based therapeutics [5,6], metal-based nanoparticles, etc. have been developed for the treatment of infectious diseases. Metal-based nanoparticles are characterized by small sizes between 10–100 nm which accounts for their good interaction with biomolecules within the cell and on the cell surface. Their high surface area promotes cell permeability [7]. They can also be tailored by conjugating selected ligands, proteins, antibodies, drugs and enzymes which have specific binding activity to selected target cells, thereby improving their targeted drug delivery capability and therapeutic efficacy at the pathological site [7]. Conjugation of drugs, antibodies, proteins etc. onto metal nanoparticles protect them against the body’s immune system, thereby extending their blood circulation time. Metal nanoparticles have good physico-chemical properties and surface charges [7]. These unique properties make metal nanoparticles potential therapeutics for the treatment of infectious diseases. Metal-based nanoparticles designed for biomedical applications must meet a series of conditions, such as being stable and not aggregate, biocompatible, selective to target cells/tissues, non-toxic and affordable. This review will focus on metal-based nanoparticles that are potential therapeutics for the treatment of infectious diseases (i.e., parasitic, viral and bacterial infections) and their biological efficacy (in vitro and in vivo).

## 2. Bacterial Infections

Bacterial infections can be treated, however, their treatment is hindered by drug resistance resulting in a worldwide public health threat. Their ability to adhere to host cells results in their colonization and they have also developed various molecular strategies which enhance their adhesion to the host cells [8]. One of the molecular strategies includes hair-like organelles on the surface of the bacteria known as pili, via which the bacteria bind to the host cells. Hair-like organelle structures are found in selected Gram-negative and Gram-positive bacteria [8,9,10]. Some bacteria can modify their pili resulting in their detachment from their original site of the colony to selected sites which are far away from this original site [8,11]. Another molecular strategy for bacterial adhesion to host cells is by the formation of biofilms that protect bacteria in a hostile environment [8,12]. Biofilm formation is reported to occur in three main stages, namely attachment to a host cell surface, proliferation and formation of the biofilm structure and detachment [13]. The biofilms provide adhesion between bacterial cells and facilitate the formation of multi-layered biofilms. The biofilm consists of components such as proteins, exopolysaccharides, eDNA, etc. These components provide protection from antibiotics and resistance to antimicrobial agents [13]. There are other molecular strategies via which bacteria adhere to the host cell.

**Figure 1:**
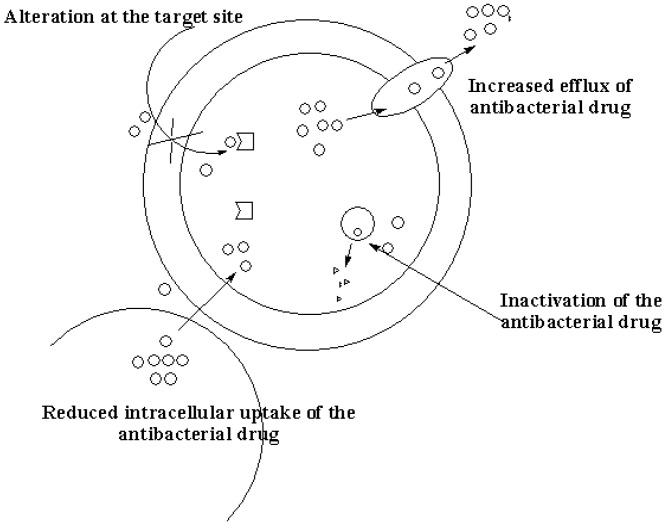
Mechanism of resistance of bacteria.

Detailed information is reported by Pizarro-Cerdá and Cossart [10]. The general mechanism of resistance of bacteria to antibacterial agents are reduced binding ability of the antibacterial agents resulting from alteration at the target site, inactivation of the antibacterial agent, increased efflux of the antibacterial agents due to reduced intracellular uptake of the antibacterial agent and reduced antibacterial activity resulting from modified metabolic pathways (Figure 1) [14,15,16,17]. Biofilm resistance to antibiotic treatment reported in vitro has been attributed to the biofilm acting as a diffusion barrier for selected antibiotics, the biofilm minimizing sensitivity to antibiotics and higher expression of specific protective molecules in the biofilm mode of growth that may result in antibiotics promoting the expression of these protective mechanisms [13]. Biofilm production by some bacteria inhibits penetration of antimicrobial agents [18,19,20]. There are several reports on metal-based drugs with enhanced antibacterial activity. This section will focus on the aforementioned classes of metal-based antibacterial compounds with antibacterial activity as discussed below.

### 2.1. Metal-Based Nanoparticles with Antibacterial Activity

Metal-based nanoparticles are characterized by small size and high surface area [21]. Their size, surface charge, and shape all influence their cellular uptake. The functionalities present on the surface of some metal nanoparticles enhance their cellular interactions. Nanoparticles exhibit antibacterial activity resulting from their ability to produce reactive oxygen species that damage the bacteria and their ability to bind to DNA or RNA, thereby hindering microbial replication processes [21,22]. Due to their small size, they can cross membrane boundaries and can be easily absorbed into the bloodstream [23]. Over the years, different types of metal-based nanoparticles have been reported by several researchers. However, some of them are still very new and their mode of action is not known. The mechanism of toxicity of metal nanoparticles on bacteria varies. Most metals are toxic to all cell types, however, they can discriminate between bacteria. Metal-based nanoparticles that have been reported with antibacterial activity include silver, iron, iron oxide, copper oxide, zinc oxide, aluminum oxide, titanium dioxide, gold and gallium nanoparticles.

#### 2.1.1. Silver-Based Nanoparticles

**Figure 2:**
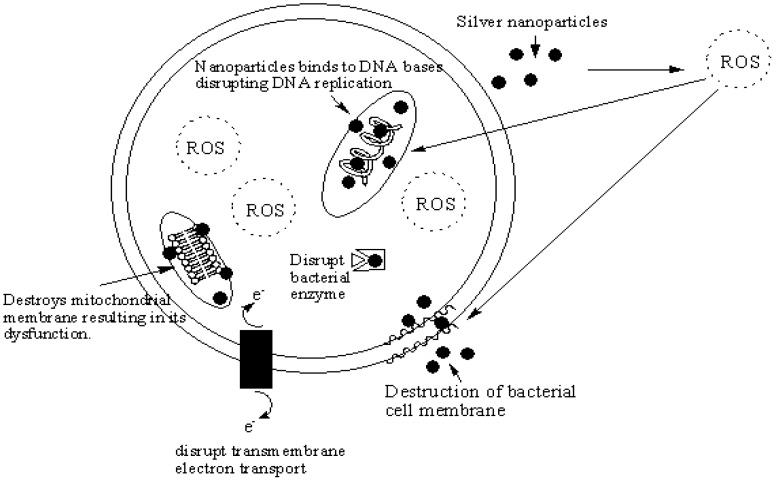
Mode of action of nanoparticles on bacteria.

Several researchers have reported the antibacterial activity of metal-based nanoparticles (Table 1). Silver nanoparticles interact with bacteria and release silver ion resulting in deactivation of the cellular enzymes, hindered membrane permeability and cell death [24,25]. Several researchers have proposed explanations for the antibacterial activity of silver nanoparticles (Figure 2).

It has been reported that silver nanoparticles penetrate bacterial cell walls, resulting in structural damage to the cell walls and cell death [26,27]. Others reported that silver nanoparticles produce free radicals that destroy the cell membrane, resulting in cell death [27,28]. Some other researchers have proposed that the silver nanoparticles release silver ions which interact with the thiol groups of many vital enzymes of the bacteria, thereby inhibiting several functions in the cell [27,29]. Silver nanoparticles have also been proposed to interact with the sulfur and phosphorus atoms of DNA, inhibiting DNA replication of the bacteria, thus causing cell death. Silver nanoparticles alter the phosphotyrosine profile of bacterial peptides, resulting in inhibition of signal transduction and inhibition of cell growth [27,30] (Figure 2).

**Figure 3:**
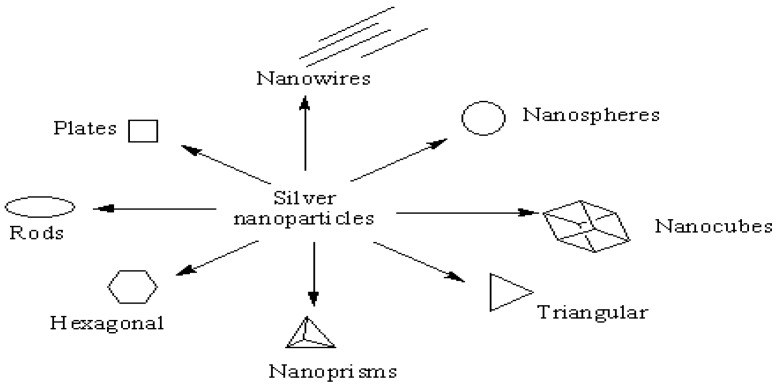
Nanoshapes of silver nanoparticles.

The shapes and the sizes of the nanoparticles of silver have been reported to influence their antibacterial effects. Different shapes of silver nanoparticles have been reported, such as nanoplates, nanospheres, nanorods, nanoprisms, nanocubes (Figure 3). Raza et al., developed triangle- and sphere-shaped silver nanoparticles of varied sizes by wet chemical routes. The antibacterial activity of the nanoparticles against *P. aeruginosa* bacteria revealed that smaller nanosized silver particles were more effective than larger sized silver nanoparticles because of their large surface area. The spherical shaped nanoparticles exhibited enhanced antibacterial activity than the triangle-shaped nanoparticles [31]. In a report by Pal et al., triangle-shaped nanoparticles exhibited high antibacterial activity than the spherical and rod shaped silver nanoparticles [32]. Similar findings were reported by Dong et al., in which triangular-shaped silver nanoparticles exhibited enhanced antibacterial activity [33]. The enhanced antibacterial effect was attributed to high-atom-density facets and interaction of the facets with the surface of the bacteria [34], suggesting that vertexes of the triangular-shaped nanoparticle results in easy penetration of the nanoparticles into the cells. Sadeghi et al., prepared hexagonal, nanorods and nanoplates-shaped silver nanoparticles [35]. Hexagonal-shaped silver nanoparticles were effective against *S. aureus* and *E. coli* when compared to nanorod- and nanoplate-shaped silver nanoparticles. However, the antibacterial effect on *E. coli* was reduced when compared to *S. aureus* and this difference in the antibacterial effects is as a result of the composition of the cell wall [36]. Hong et al., prepared nanocube, nanospheres and nanowire-shaped silver nanoparticles. Their antibacterial activity was influenced by their surface area, effective contact area and facet reactivity. Nanocube-shaped silver nanoparticles were reported to exhibit the highest antibacterial activity [37]. Silver nanoparticles have been reported to be effective for the treatment of bacterial infections such as tuberculosis, gonorrhea, chlamydia, syphilis and urinary tract infections.

Tuberculosis is an infectious disease that affects the lungs and it is caused by the bacterium *Mycobacterium tuberculosis.* The treatment of tuberculosis is hampered by drug resistance, resulting from the usage of antibiotics over a long period of time. Metal-based nanoparticles have been reported to have the potential to overcome drug resistance. Jafari et al., mixed silver and zinc oxide nanoparticles [38]. Silver nanoparticles alone exhibited low cytotoxic effects and did not inhibit the growth of *Mycobacterium tuberculosis* in vitro. However, mixing silver and zinc oxide nanoparticles at selected ratios resulted in potent antibacterial activity against *Mycobacterium tuberculosis* [38]. Praba et al., reported silver nanoparticles that exhibited growth-inhibitory effects of *M. tuberculosis* which was dependent on the concentration of the nanoparticles. A concentration of 25–50 mM of the nanoparticle inhibited the growth of *M. tuberculosis* [39]. Singh et al., reported comparative studies of silver and gold nanoparticles as anti-mycobacterial agents. Silver nanoparticles inhibited 90% of the mycobacterial growth at a concentration of 3 μg/mL [40]. Silver nanoparticles were selective towards mycobacteria, indicating their potential as therapeutics for the treatment of tuberculosis.

Apart from tuberculosis, silver nanoparticles have also been evaluated as potential therapeutics for the treatment of sexually transmitted diseases such as chlamydia, gonorrhoea and syphilis. Chlamydia is a sexually transmitted infection caused by *Chlamydia trachomatis* which usually induce severe inflammatory responses. Yilma et al., prepared silver-polyvinylpyrrolidone nanoparticles with good anti-inflammatory activity [41]. In vitro studies on a mouse J774 macrophage model of *C. trachomatis* infection revealed that the nanoparticles controlled inflammatory mediators triggered by *C. trachomatis* infection. The nanoparticles also inhibited cytokines and chemokines produced by the infected macrophages [41]. The nanoparticles were very mobile and stable with no aggregation. Silver nanoparticles have also been reported to be effective for the treatment of gonorrhea and syphilis because of their ability to interact with the bacteria cell membrane, resulting in cell death [42].

Silver nanoparticles have also been employed for the treatment of urinary tract infections. Jacob et al., reported the effect of silver nanoparticles against urinary tract infections caused by *P. aeruginosa* and *Enterobacter* [43]. The mechanism of action of silver nanoparticles was suggested to be by the attachment of the nanoparticles to the surface of the cell membrane and hindering the respiration functions of the cell, thereby disrupting the ATP production and DNA replication of the bacteria [43]. The incorporation of silver ions onto urinary catheter has also been reported to result in reduced risk of urinary tract infections [44,45]. The Bardex IC and Dover IC are catheters coated with a silver-alloy layer. The silver ions are released from the catheter to the surrounding tissues, thereby preventing urinary tract infections [46]. However, several studies have proved that silver coated catheters do not prevent urinary tract infections [47,48].

Silver nanoparticles have also been used in combination with selected antibiotics for the treatment of bacterial infections, resulting in enhanced therapeutic efficacy. Wan et al., combined silver nanoparticles with the antibiotics polymixin B and rifampicin for good synergistic effects in the treatment of *Acinetobacter baumannii* infections which are associated with hospital-acquired infections [49]. In vivo studies showed that the combination of nanoparticles with either of the antibiotics resulted in better survival ratios in *A. baumannii*-infected mouse peritonitis model. The proposed mechanism of the combination was rapid via permeability of the nanoparticles and via the outer cell membrane onto the bacteria resulting in cell death [49]. Li et al., reported the synergistic effect against *Escherichia coli* of combining amoxicillin and silver nanoparticles. The effect was attributed to chelation between selected functionalities on amoxicillin with the nanosilver particles, resulting in a formulation with potent antibacterial activity [50]. The functionalities included the hydroxyl- and amido-groups on the amoxicillin. Deng et al., investigated the synergistic effects of combining silver nanoparticles with four classes of antibiotics, namely β-lactams, quinolones, aminoglycosides and polypeptides against the drug-resistant bacteria *Salmonella typhimurium* [51]. Three classes of antibiotics showed synergistic growth inhibition against the *Salmonella* bacteria when combined with silver nanoparticles. The synergistic effects of the three classes of antibiotics were attributed to complexes formed between the antibiotics and the silver nanoparticles, resulting in the release of a high concentration of silver ion on the bacteria cell wall, thereby inhibiting bacterial growth [51], whereas the β-lactam class of antibiotics did not show any synergistic effects when combined with silver nanoparticles because of their inability to form such complexes. Smekalova et al., investigated the synergistic effects of combining silver nanoparticles with antibiotics such as gentamicin and penicillin against animal bacterial infections [52]. Silver nanoparticles combined with penicillin showed the highest antibacterial effects against *Actinobacillus pleuropneumoniae*, *A. pleuropneumoniae* and *Pasteurella multocida* [52]. The synergistic effects revealed the selectivity of the nanoparticles towards selected bacteria when combined with antibiotics. Akram et al., reported the antimicrobial effects of combinations of silver nanoparticles with visible blue light and antibiotics, namely amoxicillin, azithromycin, clarithromycin, linezolid, and vancomycin, against methicillin-resistant *Staphylococcus aureus* (MRSA) [53]. The antimicrobial activity of the combination of silver nanoparticles with light and antibiotics against MRSA isolates was enhanced with the highest antimicrobial activity when azithromycin or clarithromycin was included in the combination. The combination resulted in good synergistic antibacterial effects when compared to the antibiotic combined with either light or silver nanoparticles. The possible mechanism for the synergistic effect was the production of cytotoxic reactive oxygen species light by the visible blue light resulting in thermal destruction of the bacteria and the release of silver ions resulting in the destruction of the cell membrane of the bacterial [53]. Thirumurugan et al., evaluated the effects of combining silver nanoparticles with antibiotics such as cefazolin, mupirocin, gentamycin, neomycin, tetracycline and vancomycin against *Staphylococcus aureus, Pseudomonas aeruginosa* and *Escherichia coli* [54]. The synergistic antibacterial effect was proposed to be as a result of Reactive Oxygen Species (ROS) generated membrane cell damage [54]. Balaji et al., reported the synergistic effect of cephalexin, an antibiotic conjugated onto silver nanoparticles against *E. coli* and *S. aureus* [55]. The good synergistic effects of the formulation on Gram negative bacteria were proposed to be due to their ability to bind to the cell wall resulting in the destruction of the outer cell membrane. Destruction of the outer cell membrane resulted in cephalexin binding to the peptidoglycan structure and hence cell death [55].

Silver nanoparticles have also been prepared using plant extracts. Plant extracts contain reducing agents such as the flavonoids, carbohydrates, steroid, sapogenins, and polyphenols which are responsible for the formation of stable silver nanoparticles. Some of the plant extracts used include: *Parkia speciosa Hassk* pods [56], neem leaves [57,58,59], *Catharanthus roseus* (*C. roseus*) (L.) *G. Don*, [60], aloe vera [61], apple extract [62], phlomis [63], *Lycopersicon esculentum* [64], buchu [65], *Ocimum sanctum* [66] etc. These nanoparticles were reported to exhibit antibacterial activity.

Silver nanoparticles have also been prepared by biological methods using virus, bacteria, and fungi. The biological method of preparing silver nanoparticles offers several advantages such as: it is environmentally friendly, can be scaled up for large-scale synthesis and can control the nanostructural topography of metal ions. The antibacterial effects of these silver nanoparticles have also been reported by several researchers [67,68,69,70,71,72,73].

Based on the different biological results of silver nanoparticles in vitro and in vivo, the antibacterial activity is dependent on several factors such as the size, shape, concentration and method of preparation. The synergistic effect of antibiotics when combined with silver nanoparticles against various strains of bacteria further revealed the potential of silver nanoparticles as an antibacterial agent. The synergistic effects of silver nanoparticles when used in combination with antibiotics is dependent on the chelation of nanoparticles with the functionalities on the antibiotics suggesting that the nanoparticles act as drug carriers.

#### 2.1.2. Iron Oxide-Based Nanoparticles

Other metal-based nanoparticles include iron and iron oxide nanoparticles that have been reported by some researchers (Table 2). The mechanism of action of iron oxide nanoparticles has also been reported by several researchers. Prabhu et al., prepared iron oxide-based nanoparticles with good antibacterial activity on *E. coli* and *P. vulgaris* when compared to *S. aureus* bacterial strains [74]. The antibacterial activity was as a result of the sensitivity of the bacteria. Similar findings were reported by other researchers [75,76,77,78]. The antibacterial activity of iron oxide nanoparticles is via oxidative stress generated by ROS, resulting in the damage of the proteins and DNA in the bacteria [79]. Behera et al., prepared iron oxide nanoparticles that were effective against Gram-positive and Gram-negative bacteria [80]. The antibacterial activity of the nanoparticles was dependent on the concentration and selective towards Gram-positive bacteria. Ismail et al., prepared magnetic iron oxide nanoparticles by pulsed laser ablation [81]. The nanoparticles inhibited growth of *Staphylococcus aureus*, *Escherichia coli*, *Pseudomonas aeruginosa* and *Serratia marcescens*. Their antibacterial activity was influenced by their preparation conditions [81]. Arakah et al., prepared iron oxide nanoparticles coated with chitosan biomolecules and their antibacterial activity against a Gram positive (*Bacillus subtilis*) and a Gram negative bacterium (*Escherichia coli*) were evaluated [82]. The positively charged iron oxide nanoparticles exhibited higher antimicrobial activity than the negatively charged iron oxide nanoparticles. It was hypothesized that the interaction between negatively charged iron oxide nanoparticles and bacteria was weak due to dominant electrostatic repulsion at the interface resulting in non-attachment of the nanoparticles on the bacterial cell. However, at higher concentration, the negatively charged iron oxide nanoparticles exhibited antimicrobial activity, suggesting ROS production [82]. Irshad et al., prepared iron oxide nanoparticles using *Punica granatum* peel extract [83]. The size, morphology and antibacterial activity of the nanoparticles was influenced by the concentration of the extract used. The highest concentration produced nanoparticles with strong antibacterial activity against *Pseudomonas aeruginosa* [83]. Aparicio-Caamaño et al., evaluated the synergistic effect of combining iron oxide nanoparticles with erythromycin against *S. pneumonia* [84]. The combination inhibited the bacterial growth and viability [84]. The presence of iron oxide nanoparticles enhanced the entry of erythromycin into the bacteria revealing that nanoparticles act as carriers by delivering the drug to the bacteria resulting in inhibition of bacteria growth [84]. Tran et al., reported the effects of iron oxide nanoparticles on *Staphylococcus aureus* [85]. The nanoparticles inhibited *S. aureus* growth at a high concentration of 3 mg/mL, indicating that the antibacterial activity was influenced by the concentration [85]. Ibaraj et al., prepared chitosan coated iron nanoparticles that inhibited the growth of *Escherichia coli* and *Salmonella enteritidis* [86]. The nanoparticles generated reactive oxygen species leading to lipid peroxidation, DNA damage and protein oxidation. The positively charged surface of the nanoparticles interacted strongly with the negatively charged cell membranes via electrostatic interaction resulting in disruption of bacterial functions [86]. However, Massadeh et al., reported that iron oxide nanoparticles are not good antibacterial agents even when combined with ciprofloxacin [87]. The poor antibacterial activity of the nanoparticles suggested that their interaction with ciprofloxacin hindered the absorption of the nanoparticles on the bacterial cell. The interaction of the nanoparticles with ciprofloxacin may also have interfered with ciprofloxacin activity on bacterial DNA inside the bacterial cell [87]. Ahmad et al., prepared iron oxide nanoparticles via co-precipitation method and then modified with *Ocimum sanctum* leaf extract [88]. The nanoparticles were hexagonal in shape. The modification of the nanoparticles with the leaf extract resulted in a strong inhibition of *S. aureus* [88]. Iron oxide nanoparticles have been prepared using other plant extracts. Their shapes varied from spherical [89,90,91], chain shaped [92], cubic [93], irregular [94,95,96], hexagonal [97] and rock shaped [97].

The antibacterial activity of iron oxide nanoparticles is dependent on factors such as concentration, preparation methods, the type of charge on the nanoparticles, modification of the nanoparticles and shapes. They are highly selective and their mode of action on bacteria is reported to be attributable to the generation of reactive oxygen species leading to lipid peroxidation, DNA damage, protein oxidation and interaction with bacteria cell membranes via electrostatic interaction resulting in disruption of bacterial functions. However, the poor antibacterial activity of the iron oxide nanoparticles when combined with ciprofloxacin revealed that iron oxide nanoparticles’ interaction with the antibiotics hindered the absorption of the nanoparticles on the bacterial cell and disrupted the antibacterial activity of ciprofloxacin on the bacterial DNA inside the bacterial cell. This report suggest the need for more studies so as to fully understand the interaction of iron nanoparticles with antibiotics.

#### 2.1.3. Copper Oxide Nanoparticles

Copper oxide nanoparticles also exhibit antibacterial activity. Ahamed et al., reported copper oxide nanoparticles that were effective against a wide range of bacterial strains. However, the nanoparticles were highly active towards *E. coli* and *E. faecalis* and less active on *K. pneumonia* [98]. Pandey et al., developed copper oxide nanoparticles, namely copper oxide nanorods and multi-armed nanoparticles, by wet and electrochemical routes. The nanoparticles exhibited good antibacterial activity which was attributed to their shapes. The multi-armed nanoparticles were characterized by tapered spears, suggesting that they were in contact with the bacterial cell, they can penetrate easily into the bacterial cell, resulting in cell death. The nanoparticles killed the cells by destroying the cell membranes. Excellent bactericidal activity against Gram-negative *E. coli* bacteria was observed in which 2.3 × 10^7^ CFU/mL cells were killed within 2 h of exposure to 1 mg/mL copper oxide nanorods while 1.4 × 10^7^ CFU/mL cells were killed within a period of 30 min of exposure to 0.5 mg/mL copper oxide multi-armed nanoparticles [99]. The antibacterial activity of copper oxides is reported to be due to lipid peroxidation, generation of reactive oxygen species, protein oxidation and DNA degradation in bacteria cells [100]. Other researchers have reported similar findings in which the antibacterial activity of the nanoparticles was dependent on the particle sizes [101,102]. The smaller the particle sizes, the higher the antibacterial activity. Alswat et al., prepared copper oxide nanoparticles by green co-precipitation method [103]. The nanoparticles exhibited spherical shapes with high antibacterial activities against *Bacillus subtilis and Salmonella choleraesuis*, which was influenced by the good dispersion of nanoparticles on the zeolite surface [103]. Rani et al., reported copper oxide nanoparticles prepared by a reverse micelle technique. The antibacterial activity of the nanoparticles against *K. pneumoniae*, *S. typhimurium*, and *E. aerogenes* was significant, revealing that the nanoparticles penetrated the cell membrane, resulting in inhibition of bacterial cell growth and multiplication [104]. Hseuh et al., evaluated antimicrobial properties of copper oxides nanoparticles against different strains of *Staphylococcus aureus*. The release of copper (II) ions from the nanoparticles penetrated the bacterial cells [105]. The nanoparticles altered the reductase activity of the bacterial and also destroyed the cell membrane of the bacteria [105]. Azam et al., reported good antibacterial activity of copper oxide nanoparticles against Gram-positive (*B. subtilis* and *S. aureus*) and Gram-negative (*E. coli* and *P. aeruginosa*) bacteria [106]. The antibacterial activity of the nanoparticles was dependent on the particle size. Small sized nanoparticles exhibited good antibacterial activity. However, the nanoparticles were toxic to *E. coli* regardless of the sizes of the nanoparticles [106]. Das et al., reported the antimicrobial activity of copper oxide nanoparticle against *Escherichia coli* and *Pseudomonas aeruginosa*. The antibacterial activity of the nanoparticles was attributed to the interaction of the nanoparticles with the membrane of the bacteria [107]. Other researchers have reported the antibacterial activity of copper oxide nanoparticles [108,109,110,111,112,113]. Meghana et al., further revealed that cuprous oxide and copper oxide nanoparticles toxicity towards *E. coli* followed different mechanisms. Cuprous oxide antibacterial activity was influenced by its binding to bacteria protein, resulting in the inactivation of fumarase A which was not observed in copper oxide nanoparticles. Copper oxide nanoparticles produce significant amounts of ROS when compared to cuprous oxide nanoparticles [114]. The different mode of action of the nanoparticles was therefore influenced by their oxidation numbers [114]. Copper oxide-based nanoparticles are thus effective against Gram-positive and Gram-negative bacteria. Their antibacterial activity is reported to be attributed to generation of reactive oxygen species, protein oxidation, lipid peroxidation, destruction of cell membrane and DNA degradation in bacteria cells. Their antibacterial activity is influenced by their shapes, oxidation number and particle size.

#### 2.1.4. Zinc Oxide Nanoparticles

Zinc oxide nanoparticles exhibit good antibacterial activity against a wide range of bacteria (Table 3). Reddy et al., developed zinc oxide nanoparticles with good antibacterial activity on *Klebsiella pneumonia* that causes respiratory infection by a precipitation method [115]. The nanoparticles acted by destroying the bacterial cell wall membrane. The nanoparticles also inhibited invasion internalization by non-phagocytic cells [115]. Raghupathi et al., reported zinc oxide nanoparticles with antibacterial activity which was dependent on the size of the nanoparticles [116]. Hsueh et al., investigated the effects of zinc oxide nanoparticles on the growth of *B*. *subtilis* [117]. The inhibition effects on the growth of *B*. *subtilis* was dependent on the concentration of the nanoparticles. The accumulation of nanoparticles in the cytoplasm or on the outer membranes of the bacteria resulted in cell death [117]. Narasimha et al., reported zinc oxide nanoparticles prepared by a chemical method. The antibacterial effect against clinical isolates of *Staphylococcus aureus* was excellent [118]. Zinc oxide nanoparticles are reported to act as antibacterial agents by altering the permeability of the membrane and inducing oxidative stress resulting in bacterial cell death [118,119]. Other researchers reported similar findings [120,121,122,123]. Mirhosseini et al., reported that the antibacterial activity of zinc oxide nanoparticles was dependent on the concentration, the antibacterial effect was higher against the Gram-positive (*S. aureus*) than the Gram-negative (*E. coli*) bacteria. However, uniform distribution of the nanoparticles was crucial for enhanced antibacterial efficacy [124]. Liu et al., reported similar findings in which zinc oxide nanoparticles were effective against *E. coli*. The inhibitory effect increased with increase in the concentration of the nanoparticles. The nanoparticles damaged the bacterial cell membrane, resulting bacterial cell death [125]. Seil et al., investigated the antibacterial effects of zinc oxide nanoparticles against *S. aureus* when combined with ultrasound stimulation. The antibacterial effect of zinc oxide nanoparticles increased with a decrease in diameter. The antibacterial activity of the nanoparticles was further enhanced when combined with ultrasound stimulation which was attributed to a significant generation of hydrogen peroxide [126]. Rago et al., investigated the antibacterial effects of zinc oxide microrods and nanorods against Gram-positive bacteria. Both formulations exhibited significant antibacterial activity, however, the antibacterial activity of the nanorods was higher and more effective. No ROS generation was observed but the damage of the bacterial cells was high in *B. subtilis* cells when compared to *S. aureus*, suggesting that the morphology of the cells influenced the antibacterial activity of the formulation [127]. Voicu et al., coated zinc oxide nanoparticles with gentamicin, and its antibacterial effects against *Escherichia coli*, *Pseudomonas aeruginosa*, *Staphylococcus aureus*, *Bacillus cereus* and *Listeria monocytogenes* were significant [128]. Stan et al., synthesize zinc oxide nanoparticles with excellent antibacterial activity using aqueous extracts of *P. crispum* [129]. The antibacterial activity of nanoparticles was enhanced when compared to nanoparticles prepared by chemical synthesis. The enhanced antibacterial effects of the nanoparticles prepared using plant extract was attributed to zinc vacancy-interstitial zinc complexes and oxygen vacancies [129]. Zinc oxide nanoparticles prepared by green method have been reported to exhibit enhanced antibacterial effects against selected strain of bacteria when compared to nanoparticles prepared by chemical method [130,131]. The difference in antibacterial activity is attributed to the particle size of the nanoparticles. The mechanism of antibacterial action of zinc oxide nanoparticles is attributed to damage of bacterial cell, the release of zinc (II) ions and ROS formation [130,131].

Zinc nanoparticles are effective against bacteria when used alone, in combination with antibiotics or when used in combination with ultrasound stimulation. Their antibacterial activity is influenced by their particle size, shapes, method of preparation and concentration. The mode of action of the nanoparticles is by accumulation of in the cytoplasm or on the outer membranes of the bacteria and inducing oxidative stress resulting in bacterial cell death.

#### 2.1.5. Aluminium Oxide-Based Nanoparticles

Aluminium oxide nanoparticles’ antibacterial activity is attributed to the attachment of the nanoparticles to the cell surface of the bacteria, resulting in cell death [132]. There are few reports on the antibacterial activity of aluminium oxide nanoparticles (Table 3). Jalal et al., prepared aluminium oxide nanoparticles using leaf extract of *Cymbopogon citratus*. The nanoparticles penetrated *Candida* cells, disrupting the morphological and physiological activity of the cells, thus resulting in cell death [133]. Ansari et al., prepared aluminium oxide nanoparticles using leaf extracts of lemongrass [134]. Their antibacterial activity against extended-spectrum β-lactamases and metallo-β-lactamases of clinical isolates of *P. aeruginosa* was significant [134]. Other researchers also reported similar antibacterial activity of aluminium oxide nanoparticles against Gram-positive and Gram-negative bacteria [135,136]. The antibacterial activity of the nanoparticles was suggested to be due to a combination of factors such as the cationic size of nanoparticles, reactive oxygen species formation and self-promoted uptake mechanism of the nanoparticles across the outer membranes of bacteria resulting in cell death [135]. Sadiq et al., reported that aluminium oxide nanoparticles do not exhibit strong antibacterial activity, even at high concentrations, suggesting that the nanoparticles have mild toxicity toward selected strains of bacteria [132]. Aluminium oxide nanoparticles are very stable over a wide range of temperatures. Their antibacterial activity is attributed to their interaction with the cell membrane, resulting in distortion of the cell membrane and bacterial cell death. However, the antibacterial effects of aluminium oxide nanoparticles is mild and dependent on the concentration of the nanoparticles and there is a need for a good understanding of its mechanism of action on bacteria.

#### 2.1.6. Gold-Based Nanoparticles

Gold-based nanoparticles have also been reported to be effective antibacterial agents (Table 4). Mohamed et al., prepared gold-based nanoparticles which were effective against *Corynebacterium pseudotuberculosis*, a bacterial infection that affects sheep [137]. The antibacterial activity of gold nanoparticles is due to the penetration ability of the nanoparticles via the cell wall [137]. Zhou et al., reported gold-based nanoparticles that inhibited growth of *E. coli* [138]. Li et al., reported functionalized gold nanoparticles effective against Gram-negative and Gram-positive uropathogens and multi-drug resistant pathogens. Cationic hydrophobic gold nanoparticles were effective at disrupting the integrity of bacterial membrane and causing toxicity to bacterial cells. They exhibited mild toxicity to mammalian cells [139]. Other researchers reported the antibacterial efficacy of gold nanoparticles [140,141,142,143,144,145,146,147,148,149,150,151,152]. The antibacterial activity of gold nanoparticles against gram-positive and gram-negative bacteria is different because of the structure of the bacteria membrane suggesting the need for higher dose of the nanoparticles. It was reported that gold nanoparticles antibacterial activity occur due to reduced adenosine triphosphate synthase activities which disrupt the metabolic process and a decline of the subunit of the ribosome for tRNA binding, thus resulting in a collapse of the biological mechanism [142]. The high surface area enhanced the direct interaction of the nanoparticles with the bacteria. The nanoparticle interfered with the bacteria protein and cytoplasm, causing cell death [142,143]. Gold nanoparticles combined with antibiotics have resulted in enhanced antibacterial activity [153,154,155,156,157,158,159,160,161,162]. Selected antibiotics have been used such as gentamicin [153], antibodies [154,155], vancomycin [156,157], ampicillin [158], streptomycin [158], kanamycin [158] and levofloxacin [159]. The prepared gold nanoparticles did not exhibit antibacterial activity, however, they acted as a drug carrier for the delivery of gentamicin due to their large surface area. The antibacterial activity of nanoparticles combined with gentamicin was not significant when compared to gentamicin alone [153]. Gold particles conjugated with anti-protein A antibodies for selective antibacterial activity resulted in enhanced cell permeability and irreparable damage to the cell membrane [154]. The combination of anti-protein A antibody, and gold nanorods with laser energy resulted in reduced MRSA cells viability in vitro and in vivo [155]. Gold nanoparticles combined with vancomycin and illuminated with near infra-red light resulted in selective binding to the cell wall of selected bacteria. This approach was effective for drug resistant Gram-positive bacteria, Gram-negative bacteria and antibiotic-resistant bacteria [156,157]. Saha et al., combined gold nanoparticles with ampicillin, streptomycin and kanamycin [158]. The combination was effective against *E. coli DH5α*, *Micrococcus luteus* and *Staphylococcus aureus*. Combining gold nanoparticles with streptomycin or kanamycin showed significant antibacterial activity when compared to gold nanoparticles combined with ampicillin [158]. Conjugating the antibiotics to the nanoparticles resulted in enhance antibacterial activity when compared to the free antibiotics suggesting that gold nanoparticles enhanced the interaction of the formulation with the bacterial cell [158]. The mild antibacterial activity of gold nanoparticle-ampicillin was due to its poor stability [158]. However, the free gold nanoparticles did not exhibit antibacterial activity. A combination of gold nanoparticles with levofloxacin was effective against *S. aureus* and *E. coli.* The antibacterial activity was due to the disruptions of the cell wall resulting in cell death. The formulation was highly stable and was able to target bacteria selectively [159].

Free gold nanoparticles are ineffective against bacteria. However, when modified they act as drug carriers and their combination with antibiotics results in synergistic antibacterial activity. Gold nanoparticles are very stable and their ability to interact with bacteria cells make them potential antibacterial agent. Their mode of action on bacteria is attributed to reduced adenosine triphosphate synthase activities thereby disrupting the metabolic process and a decline of the subunit of the ribosome for tRNA binding, their interference with bacterial proteins and cytoplasm and targeted mechanism in which they can target selected bacteria.

#### 2.1.7. Titanium Dioxide-Based Nanoparticles

Titanium dioxide have also been reported to be a potential antibacterial agent (Table 4). Jesline et al., reported the efficacy of titanium dioxide nanoparticles against biofilm-producing methicillin-resistant *S. aureus*. The antibacterial efficacy of titanium dioxide was attributed to the electromagnetic attraction between the bacteria and the nanoparticles resulting in cell death [160]. Thomas et al., reported the antibacterial efficacy of titanium dioxide nanoparticles against bacteria that cause dental plaque [161]. Santhoshkumar et al., prepared titanium dioxide from aqueous leaf extract of *Psidium guajava* with good antibacterial effects [162]. Other researchers reported the antibacterial activity of titanium dioxide nanoparticles [163,164]. Titanium dioxide nanoparticles’ antibacterial activity is attributed to their interaction with the bacteria cell upon which photocatalytic action results in enhanced cell permeability, causing accelerated photo-oxidation of the intracellular components and cell death [165]. This process usually results in delayed bacterial activity of titanium dioxide nanoparticles [165]. Lin et al., prepared TiO_2_ NPs with smaller particle sizes which produced high contents of intracellular reactive oxygen species. The small surface area of nanoparticles resulted in membrane damage and internalization [166]. Planchon et al., reported that toxicity of titanium dioxide nanoparticles was not attributable to the adsorption of the particles onto the cell surface at selected pH [167]. However, the antibacterial effect of titanium dioxide nanoparticles is attributed to production of reactive oxygen species generation, direct interaction of the nanoparticles with the cell wall resulting in the penetration of the nanoparticles [167]. Tong et al., evaluated the toxicity of titanium dioxide nanoparticles against bacteria in natural aquatic systems. The size of nature of aggregation of the nanoparticles in combination with environmental factors enhanced its toxicity towards bacteria [168]. The cytotoxicity of the nanoparticles on *E. coli* was greater compared to the combination of the nanoparticles with light or light illumination alone [168].

Titanium dioxide nanoparticles antibacterial activity is attributed to their interaction with the bacteria cell upon which photocatalytic action results in enhanced cell permeability, causing accelerated photo oxidation of the intracellular components and cell death which is characterized by delayed bacterial activity and production of reactive oxygen species generation.

#### 2.1.8. Gallium-Based Nanoparticles

Gallium-based nanoparticles have been reported to exhibit antibacterial activity (Table 4). Gallium exhibits many similarities compared to iron metal. However, gallium ion cannot be reduced, suggesting that it can bind to the iron binding sites in the enzyme protein, thereby rendering the enzyme inactive and inhibiting bacterial growth [169]. Narayanasamy et al., reported that gallium nanoparticles reduced the growth of *Mycobacterium* for 15 days after a single drug loading [169]. Kurtjak et al., developed gallium nanospheres with good antibacterial properties against *Pseudomonas aeruginosa* [170]. This finding revealed the potential application of gallium nanoparticles to prevent infections caused by *P. aeruginosa* [170]. Choi et al., prepared gallium nanoparticles for enhanced targeting of *M*.*tb* infected-macrophages [171]. The release of gallium inhibited *M*.*tb* growth significantly in vitro. The nanoparticles promoted maturation of the phagosome, indicating their potential application as therapeutic anti-tuberculous drugs [171]. The nanoparticles inhibited iron acquisition by mycobacteria [171]. Olakanmi et al., reported that gallium nanoparticles disrupt *F. tularensis* Fe metabolism thereby inhibiting infection [172]. However, at low concentrations, the antibacterial effect of the nanoparticles was low [172].

## 3. Viral Infections

Some viral infections can be cleared from the body via the immune system. However, some are persistent and the infections can last for years such as herpes, hepatitis, HIV, etc. Presently, very few drugs can be used to hinder the spread of viral invaders, indicating that there is a pressing need to develop new drug systems which can be effective for the treatment of viral infections. The treatment of viral infections is hampered by drug resistance as a result of viral replication and extended period of exposure to the resistant strain of virus to the drug [173]. Drug resistance results in drug toxicity, severe disease and death. Examples of viral infections are HIV, herpes, cervical cancer, influenza, hepatitis etc. There are reports on the application of metal-based therapeutics for the treatment of viral infections which will be discussed below in detail.

### 3.1. HIV

**Figure 4:**
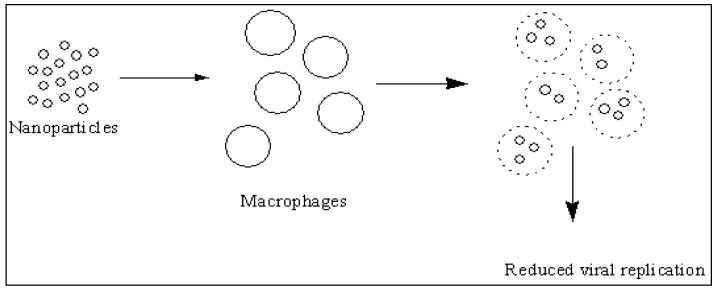
Mode of action of nanoparticles on microphages.

**Figure 5:**
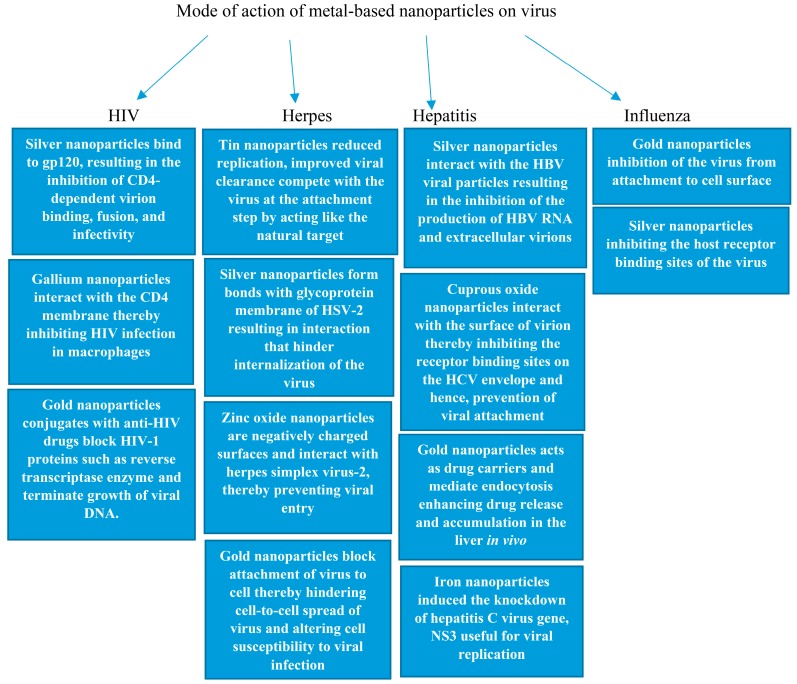
Mode of action of nanoparticles on viruses.

HIV is a viral infection with 36.7 million people living with HIV/AIDS. In 2015, it was estimated that there were 2.1 million new infections worldwide [174]. Metal-based nanoparticles (Table 5) have been found to be effective against HIV virus, resulting in decreased viral growth and replication. Examples are silver, gallium and gold nanoparticles (Figure 4). The mode of action of these nanoparticles on viruses varies (Figure 5).

#### 3.1.1. Silver Nanoparticles

Silver nanoparticles are also effective potential therapeutics for the treatment of HIV infections. Lara et al., reported the potential of silver nanoparticles in exerting anti-HIV activity at both the early stage of viral replication and at the post entry stage of the HIV-1 life cycle [175]. In the early stage, the nanoparticles acted by binding to gp120, resulting in the inhibition of CD4-dependent virion binding, fusion, and infectivity [175]. However, the antiviral mode of action of silver nanoparticles is not fully understood. There are also other reports of silver nanoparticles as potential antiviral agents effective against post-infected HIV-1 activity [176]. HIV infection of host cell occurs when gp120 binds to the CD4 receptor site on the host cell. The binding to CD4 results in a conformational change induced in gp120, whereby new binding sites for a chemokine receptor, are exposed [177]. The interaction of silver nanoparticles with gp120 glycoprotein hinders the virus from binding with host cells [177].

#### 3.1.2. Gallium Nanoparticles

Gallium nanoparticles have also been found to be effective against HIV virus. Choi et al., reported the potential of gallium nanoparticles in supressing co-infection of HIV and tuberculosis [178]. The nanoparticles interacted with the CD4 membrane, resulting in endocytosis. pH-dependent endosomal escape of the nanoparticles into the cell cytoplasm resulted in inhibition of viral protease [179]. Soto et al., prepared glucan particles loaded with gallium nanoparticles for delivery of gallium and inhibition of HIV infection in macrophages [180]. The formulation inhibited 95% HIV growth when compared to the free gallium nanoparticles. Gallium nanoparticles lack specificity and loading the nanoparticles onto carriers resulted in efficient targeted delivery of the nanoparticles to macrophages by a receptor-mediated uptake mechanism [180].

#### 3.1.3. Gold Nanoparticles

Gold nanoparticles are employed as delivery systems for enhanced efficacy of anti-HIV drugs. Free gold nanoparticles are ineffective against HIV infection. Kesarkar stabilized gold nanoparticles with amino acid l-Cysteine for enhanced cell internalization and delivery of azidothymidine against HIV-1Ba-L virus in vitro [181]. The nanoparticles exerted anti-HIV activity at early stages of viral replication. The anti-HIV activity of the nanoparticles is due to their polyanionic surface which can bind to the positively charged amino acids in the binding site of the viral envelope glycoprotein gp120. Post-entry inhibition studies further revealed that gold nanoparticles blocked HIV-1 proteins such as reverse transcriptase enzyme etc. [181,182]. Garrido conjugated gold nanoparticles with raltegravir with good HIV activity. The free gold nanoparticles did not exhibit anti-HIV activity [183]. Kesarkar coated gold nanoparticles with polyethylene glycol. The nanoparticles showed greater antiviral activity when allowed to interact with the virus. The formulations at a concentration of 2 ppm and 4 ppm were more effective in inhibiting viral entry. Gold nanoparticles acted as virus entry inhibitors and virus neutralizing agent [184]. Chiodo et al., reported carbohydrate-coated gold nanoparticles conjugated with nucleoside reverse transcriptase inhibitors namely, abacavir and lamivudine [185]. The nanoparticles inhibited HIV viral replication in vitro similar to the free drugs. The delivery of the drug from the nanoparticles inhibited viral replication thereby terminating the growth of viral DNA [185]. Bowman et al., conjugated a fragment of a potent HIV inhibitor, TAK-779, to gold nanoparticles [186]. Drug-gold nanoparticle exhibited anti-HIV activity similar to TAK-779. However, the fragment did not exhibit antiviral activity suggesting that incorporation of inactive drug onto gold nanoparticle surfaces can make the drug a potent therapeutics [186]. Peptide triazoles have been conjugated onto gold nanoparticles, resulting in potent antiviral effects against HIV-1 when compared to the free peptide triazoles [187]. Increasing the nanoparticles diameter and the density of peptide triazoles conjugated on the nanoparticle surface enhanced inhibition of infection [187].

### 3.2. Herpes

Herpes is a disease caused by herpes simplex virus HSV-1 and HSV-2. However, HSV-2 is associated with sexually transmitted diseases. Herpes simplex virus can replicate various tissues and escape anti-HSV antibodies [188]. Herpes simplex virus causes invasive cervical carcinoma [189]. The available anti-HSV drugs do not eliminate the virus and can result in serious complications such as encephalitis. There are few reports which demonstrate the application of metal-based nanoparticles for the treatment of herpes infections such as, tin nanoparticles, silver, zinc oxide and gold nanoparticles (Table 5).

#### 3.2.1. Tin Nanoparticles

Tin nanoparticles are potential anti-HIV agent. Trigilio et al., developed tin nanoparticles by flame transport synthesis with antiviral activity and studied their potential to trap HSV-1 before entry into the host cell. The nanoparticles were negatively charged and inhibited cell entry suggesting reduced replication, improved viral clearance and good antiviral effects [190]. The nanoparticles were able to compete with the virus at the attachment step by acting like the natural target [190].

#### 3.2.2. Silver Nanoparticles

Interaction between silver nanoparticles and HSV-2 have resulted in significant reduction of progeny viruses with weak cytotoxicity in vitro [191]. In vitro studies on Vero cells revealed that higher concentration of silver nanoparticles was toxic to Vero cells [191]. Virus replication was inhibited at a concentration of 100 μg/mL of silver nanoparticles. The nanoparticles formed bonds with glycoprotein membrane of HSV-2 resulting in interaction that hindered internalization of the virus. This was attributed to the interaction between the glycoprotein and a receptor [191]. Tannic acid modified with silver nanoparticles reduced HSV-2 infection vitro and in vivo [192]. The antiviral activity of the formulation was influenced by the particle size and the dose of the formulation. The nanoparticles hindered virus attachment and penetration. Smaller-sized nanoparticles were characterized by the production of cytokines and chemokines useful for anti-viral response [192]. Silver nanoparticles capped with mercaptoethane sulfonate compete for binding to cellular cell surface heparan sulfate via sulfonate end groups resulting in hindrance of viral entry into the cell and prevention of subsequent infection [193]. Polyurethane condoms coated with silver nanoparticles inhibited HSV-1 and HSV-2 infection. Nanoparticles were very stable on the condom [194]. Silver nanoparticles interaction with herpes simplex virus types 1 and 2 is influenced by their size and method of preparation [195]. They reduce viral infection by inhibiting the interaction of the virus with the cell. Smaller-sized nanoparticles inhibit viral infection significantly [195].

#### 3.2.3. Zinc Oxide Nanoparticles

Zinc oxide nanoparticles are characterized by negatively charged surfaces that can interact with herpes simplex virus-2, thereby prevent viral entry [196]. HSV-2 virus bound to the nanoparticles cannot infect cells because the dendritic cells in the vaginal lining produce antibodies that identify and destroy the infected cells thereby hindering the spread of the infection [196]. Zinc oxide micro-nano structures capped with multiple nanoscopic spikes mimicking cell induced filopodia have been reported [197]. These formulations target the virus to compete for its binding to cellular surface heparan sulfate via partially negatively charged oxygen vacancies on their nanoscopic spikes thereby inhibiting viral entry and subsequent infection. The negatively charged nanoparticles trapped the virions hindering HSV-1 infection [197].

#### 3.2.4. Gold Nanoparticle

Presently, there are limited research reports on the application of gold nanoparticles for the treatment of herpes infections. Sarid et al., invented water-soluble sulfonate-protected gold nanoparticles for the prevention herpes infections. The nanoparticles interacted with the virus by inhibiting viral attachment and penetration into the cells thereby preventing infections [198]. Baram-Pinto et al., reported gold-based mercarptoethene sulfonate nanoparticles that inhibited HSV-1 virus. The formulation was non-toxic and useful for topical application as prophylactic and therapeutic applications. The nanoparticles blocked attachment of virus to cell thereby inhibiting cell-to-cell spread of virus and altering cell susceptibility to viral infection [199].

### 3.3. Hepatitis

Hepatitis is a viral infection that affects the liver. It can be classified as hepatitis A, B, C, D, E and G. Hepatitis A is caused by an RNA virus and it is found mostly in the faeces of infected individuals. The virus spreads via the faecal-oral route. The virus may also be spread through sexual contact [200]. The infection is common in developing countries and in regions with poor sanitation [200]. Hepatitis B infection is caused by DNA virus. It is transmitted parenterally and sexually. It can be transmitted via blood transfusion or by sharing injection needles. Hepatitis C is caused by RNA virus and it is also transmitted parenterally, perinatally, sexually or when exposed to infected blood [200]. Despite the available antiviral agent for the treatment of hepatitis infections, the available drugs suffer from drug resistance, indicating that there is a need to design therapeutics that can overcome this drug resistance. Metal-based nanoparticles such as gold, silver, iron oxide and cuprous oxide nanoparticles have been found to be potential therapeutics for the treatment of hepatitis infections (Table 5).

#### 3.3.1. Silver Nanoparticles

The effects of silver nanoparticles on hepatitis B virus have been reported using silver nanoparticles with mean particle diameters of 10 nm and 50 nm [201]. In vitro anti-HBV evaluation of these particles on HepAD38 cell line revealed that nanoparticles reduced the extracellular HBV DNA formation of HepAD38 cells by over 50%. The nanoparticles interaction with the HBV viral particles resulted in the inhibition of the production of HBV RNA and extracellular virions [201].

#### 3.3.2. Iron Oxide Nanoparticles

Iron nanoparticles have been employed for targeted delivery systems for the delivery of DNAzyme for the treatment of hepatitis C [202]. The nanoparticles induced the knockdown of hepatitis C virus gene, NS3. HCV NS3 gene encodes helicase and protease which are useful for viral replication. The nanoformulation did not suffer from severe immune responses [202]. In vivo evaluation on mice showed that after administration on the animal models, the nanoparticles accumulated in the hepatocytes and macrophages in the liver suggesting their potential application for the treatment of hepatitis C [202].

#### 3.3.3. Cuprous Oxide Nanoparticles

Cuprous nanoparticles efficacy against hepatitis C have been evaluated in vitro [203]. The nanoparticles inhibited infection at a concentration of 2 µg/mL. They inhibited the entry of virus which included genotypes such as, 1a, 1b and 2a thereby hindering viral replication. The nanoparticles inhibited infection at attachment and entry stages indicating their potential in the treatment of chronic hepatitis C [203]. The nanoparticles interacted with the surface of virion thereby inhibiting the receptor binding sites on the HCV envelope and hence, prevention of viral attachment [203].

#### 3.3.4. Gold Nanoparticles

The effect of gold nanoparticles on viral load of hepatitis C virus has been reported [204]. In vitro evaluation on blood samples infected with HCV for HCV viral load revealed that a 1:1 ratio and gold nanoparticles exhibited no effects on the virus [204]. Despite the non-viral activity of gold nanoparticles, they have been employed as drug delivery systems. Hyaluronic acid-gold nanoparticles were designed for the delivery of interferon α for the treatment of hepatitis C infection [205]. The formulation was delivered to the liver by hyaluronic acid receptor mediated endocytosis enhancing the release of interferon α induced cytokine [205]. The accumulation of the drug in the liver in vivo was specific and enhanced revealing the potential of gold nanoparticles for targeted drug delivery [205].

### 3.4. Influenza

Influenza is a respiratory viral infection in human that is acute and the cause of high death rate globally especially in children, the elderly and people living with chronic diseases [206]. Metal-based nanoparticles such as gold and silver nanoparticles have been designed and reported to be effective against influenza virus (Table 5). Thiosialoside molecules have been tethered to silver and gold nanoparticles resulting in glycoclusters via artificial thioglycoside bonds to overcome high molecular weight that can limit their effectiveness. The formulation was effective against influenza. A virus [207]. Xiang et al., demonstrated the potential application of silver nanoparticles for the treatment of H3N2 influenza viruses, resulting in damage of the morphological structure of the viruses. The damage was dependent on time [208]. Mori et al., developed chitosan composites loaded with silver nanoparticles. The antiviral activity of the composites increased with increase in the concentration of silver nanoparticles [209]. The chitosan matrix reduced the interaction between the silver nanoparticles and the virions, suggesting that the interaction increased with increase in the concentration of silver nanoparticles in the composites [209]. Li et al., reported the delivery of silver nanoparticles with amantadine. The formulation hindered HINI infection by hindering reactive oxygen species accumulation and infection of the virus on the host cell [210]. Sametband et al., demonstrated the therapeutic efficacy of anionic gold nanoparticles against influenza virus [211]. The antiviral activity of the nanoparticles was by inhibition of the virus from attachment to cell surface. However, the functional group also played an importance role in the antiviral activity of the formulation [211]. Another report also revealed the anti-influenza activity of silver nanoparticles which was influenced by the size of the nanoparticle [212]. Mehrbod et al., reported that nanosilver inhibited interaction between glycoprotein knobs and antibodies. At a concentration of at 0.5 µg/mL, viral infectivity was reduced whereas pre and postpenetration were more effective (*p* < 0.05). The ability of nanosilver to target the disulfide bonds plays an important role in their antiviral activity thereby inhibiting the host receptor binding sites of the virus [213].

## 4. Parasitic Infections

Parasitic infections are caused by protozoa, helminths, etc. Some examples of parasitic infections are malaria, leishmaniasis, trypanosomiasis, schistosomiasis, etc. The treatment of parasitic infections is hampered by drug toxicity and resistance, resulting in the application of two or more selected antiparasitic drugs for enhanced therapeutic outcomes [214]. Parasitic infection such as malaria suffer from drug resistance as a result of poor treatment compliance [215].

### 4.1. Malaria

Malaria is a serious disease caused by the protozoan parasite *Plasmodium*. The major challenges in the treatment of malaria infections include drug resistance, which is attributed to poor treatment compliance, rate of mutation of the parasite, overall parasite load, efficacy of the selected drugs and the co-infection of different strains of malaria parasites [215]. There are reports on metal nanoparticles with antimalarial activity such as silver, gold, iron oxide, magnesium oxide, aluminium oxide, etc. (Table 6).

#### 4.1.1. Silver Nanoparticles

Silver nanoparticles’ antiplasmodial activity has been reported by a few researchers. The antiplasmodial activity of silver nanoparticles prepared from plant extracts is attributed to the presence of bioactive metabolites which act as anti-oxidative activity against oxidative stress induced in the host parasitized red blood cells by the malarial parasites. Mishra et al., investigated the ability of silver nanoparticles prepared from leaf extracts to inhibit the growth of *P. falciparum* in ex vivo human red blood cell culture [216]. These nanoparticles prepared from the plant extract were found to have antiplasmodial effects with IC_50_ (g/mL) of 3.75 for Amylase, 8 for Ashoka and 30 for Neem. The plant extracts did not show any activity up to 40 g/mL. The nanoparticles did not show any sign of hemolysis [216]. Jaganathan et al., prepared silver nanoparticles using *Eudrilus eugeniae* earthworms as reducing and stabilizing agent [217]. The antiplasmodial activity of the nanoparticles against chloroquine-resistant and chloroquine-sensitive strains of *Plasmodium falciparum* IC_50_ were 55.5 μg/mL and 49.3 μg/mL, respectively [217]. The inhibition effect of the nanoparticles on *P. falciparum* was higher than chloroquine. The antiplamodial efficacy of the nanoparticles was attributed to their ability to inhibit *P. falciparum* merozoite invasion into the erythrocytes [217]. Murugan et al., prepared silver nanoparticles using *C. tomentosum* and spongeweed as a reducing and capping agent [218]. The antiplasmodial activity of nanoparticles prepared from *C. tomentosum* extract against chloroquine-resistant and chloroquine-sensitive strains of *Plasmodium falciparum* (IC_50_) was 76.08 and 72.45 μg/mL, respectively [218]. Murugan et al., also synthesized silver nanoparticles using *Azadirachta indica* seed kernel extract as reducing and stabilizing agent [219]. The antiplasmodial activity IC_50_ of the nanoparticles against chloroquine-resistant and chloroquine-sensitive strains of *Plasmodium falciparum* was 86.12 and 82.41 μg/mL, respectively. In vivo antiplasmodial evaluation of the nanoparticles on *Plasmodium berghei* infected albino mice revealed moderate activity [219].

#### 4.1.2. Metal Oxide Nanoparticles

The only report on antiplasmodial activity of metal oxide nanoparticles is by Inbaneson et al., Metal oxide nanoparticles such as, Fe_3_O_4_, MgO, ZrO_2_, Al_2_O_3_ and CeO_2_ were coated with PDDS and their antiplasmodial activity against *P. falciparum* was evaluated using chloroquine and artemether as control. Antiplasmodial (IC_50_) activity of synthesised PDDS coated nanoparticles were 69.97, 67.07, 48.66, 79.66 and 60.28 for PDDS-Al_2_O_3_, PDDS-CeO_2_, PDDS-Fe_3_O_4_, PDDS-ZrO_2_ and PDDS-MgO, respectively, when compared to chloroquine and artemeter which were 19.59 and 4.09, respectively. However, the PDDS-coated metal oxide nanoparticles showed superior antiplasmodial activity than the non-PDDS-coated metal oxide nanoparticles [220].

#### 4.1.3. Gold Nanoparticles

Karthik et al., developed *Streptomyces* sp. LK-3 (JF710608)-mediated gold nanoparticles with particle size range between 5–50 nm [221]. In vivo evaluation on *Plasmodium berghei* infected mice delayed the parasitemia rise by 6% over a period of 8 days after infection. The results obtained suggest that the gold nanoparticles are potential therapeutics for the treatment of malarial [221]. Dutta et al., synthesized gold nanoparticles using leaf and bark extract of *Syzygium jambos* (L.) Alston (Myrtaceae). A [222]. Gold nanoparticles synthesized by bark and leaf extract of S. jambos showed antiplasmodial activity with IC_50_ values of 49.54 and 45.49 µg/mL against chloroquine sensitive strain *P. falciparum* and 51.63 and 49.38 µgm/L against chloroquine resistant strain of *P. falciparum* [222].

### 4.2. Leishmaniasis

Leishmaniasis is a disease caused by a protozoa parasite, *Leishmania,* transmitted by the bite of a female sandfly (*Phlebotomus* species) [223]. Poverty and malnutrition play a major role in the increased transmission of the disease. The disease is classified as visceral, cutaneous and post-kalaazar dermal leishmaniasis, mucocutaneous leishmaniasis. Mucocutaneous leishmaniasis is chronic, fatal and progressive [224]. It affects the mucous membranes of the mouth, nose, and soft palate, and result in severe midfacial mutilation [223]. Visceral leishmaniasis affects individuals with poor states of health and poor nutritional status [223]. Coinfection of a patient with human immunodeficiency virus with leishmaniasis accelerates the onset of acquired immunodeficiency syndrome by cumulative immunosuppression and by stimulating the replication of the virus [223]. In the developing countries the high cost and side effects associated with the anti-leishmanial hinders patient compliance. Some of the anti-leishmanial drugs suffer from drug resistance and some of the mechanism of resistance to some the currently used anti-leishmanial drugs include: increased efflux mechanism, decreased drug concentration inside the parasite, inhibition of drug activation, inactivation of active drug, etc. [224]. Some researchers have designed metal-based nanoparticles such as silver, gold, titanium dioxide, zinc oxide and magnesium oxide nanoparticles that have potential to overcome the mechanism of resistance of the currently used anti-leishmanial (Table 6).

#### 4.2.1. Silver Nanoparticles

Some researchers have investigated the efficacy of silver nanoparticles as anti-leishmanial agents. Ameneh et al., demonstrated that combination of UV light with silver nanoparticles resulted in inhibition of the proliferation and metabolic activity of promastigotes by 2- to 6.5-fold [225]. The combination also inhibited the survival of amastigotes in host cells significantly. In similar studies, silver nanoparticle combined with UV light resulted in good synergistic anti-leishmanial effects in vivo [225]. The nanoparticles attachment to sulfur and phosphorus groups increased its anti-leishmanial effects providing a high capacity of ROS production [225]. The toxic effects of the nanoparticles were enhanced by irradiation of UV photons resulting in the release of releasing ions [225]. Rossi-Bergmann et al., compared in vitro and in vivo antileishmanial activity of the silver nanoparticles prepared by chemical process and by biosynthesis from *Fusarium oxysporum* [226]. In vitro antipromastigote activity of *L. amazonensis* showed that chemically synthesized silver nanoparticles, biosynthesized silver nanoparticles and amphotericin B, and anti-leishmanial drug, decreased the parasite load up to 13%, 61% and 68%, respectively. The IC_50_ values were 103.5 ± 11.5 μM and 31.6 ± 8.2 μM for chemically synthesized and biosynthesized silver nanoparticles, respectively. In vivo studies on BALB/c mice further revealed that the parasitemia inhibition of the silver nanoparticles was the same with amphotericin B at lower concentration than amphotericin B [226]. The results suggested that nanoparticles can be used for the treatment of cutaneous leishmaniasis [226]. Lima et al., prepared chitosan-based silver nanoparticles with induced antileishmanial activity against promastigote forms of *Leishmania amazonensis* at minimum inhibitory concentrations between 1.69 and 3.38 µg Ag/mL [227]. The nanoparticles were active against *L. amazonensis* when compared to chitosan [227]. Ahmad et al., used phytochemicals from *Sargentodoxa cuneata* to reduce and stabilize the silver and gold ions into metallic nanoparticles with sizes between 3–8 nm. In vitro antileishmanial activity revealed the IC_50_ value of 4.37 and 5.29 μg mL^−1^ for silver and gold nanoparticles, respectively. The application of *Sargentodoxa cuneata* as a reducing and capping agent retained the biological activity of the nanoparticles [228]. Zahir et al., reported silver nanoparticles that were effective against *Leishmania* parasites with IC_50_ of 14.94 μg/mL and 3.89 μg/mL in promastigotes and intracellular amastigotes, respectively [229]. The growth inhibitory effect of synthesized nanoparticle was attributed to increased length of S phase, a reduced reactive oxygen species level with an inhibition of trypanothione/trypanothione reductase system of *Leishmania* cells [229].

#### 4.2.2. Gold Nanoparticles

Gold nanoparticles are effective against drug resistant strains of leishmaniasis. Halder et al., prepared monodispersed kaempferol-stabilized gold nanoparticles with particle size of 18.24 nm. The nanoparticles were effective against drug resistant organisms [230]. The resistance index for the nanoparticles was higher in resistant strains suggesting specific efficacy when compared to kaempferol. The nanoparticles were effective against both wild-type and drug-resistant strains. The selectivity index for the nanoparticles was lower than for amphotericin B [230]. Ahmad reported gold nanoparticles with good anti-leishmanial activity influenced by their surface macromolecules [229]. Torabi et al., assessed nanogold efficacy against cutaneous leishmaniasis [231]. In vivo studies on Iranian strain of *Leishmania* in BALB/c mice model induced by inoculation with Iranian *Leishmania major* promastigote showed that topical application of the formulation reduced amastigote number into the lesions significantly. The nanogold had therapeutic effect on cutaneous leishmaniaisis and decreased progression of the disease in BALB/c animal model [231].

#### 4.2.3. Metal Oxide Nanoparticles

Jebali and Kazemi reported the antileishmanial activity of nanoparticles when used in combination with UV/IR light [232]. Metal oxide nanoparticles such as titanium dioxide nanoparticles, zinc oxide nanoparticles and magnesium oxide nanoparticles antileishmanial activity increased from titanium dioxide nanoparticles, followed by zinc oxide and magnesium oxide nanoparticles. UV and IR light enhanced antileishmanial properties of all nanoparticles [232]. Zinc oxide have been evaluated as a potential antileishmanial agent [233,234]. Delavari et al., reported zinc oxide nanoparticles with dose dependent anti-leishmanial activity. The IC_50_ after 24 h of incubation was 37.8 μg/mL. The nanoparticles exerted cytotoxic effects on promastigotes of *L. major* via induction of apoptosis. A concentration of 120 μg/mL of ZnO nanoparticles induced 93.76% apoptosis in *L. major* after 72 h [233].

### 4.3. Helminth Infections

Helminth infections are considered neglected tropical diseases [235]. Helminth are parasitic worms which are invertebrate, elongated, round or flat bodies [235,236]. The most common helminth are intestinal nematodes, schistosomes and filarial worms [235]. Helminth infection causes morbidity and mortality [235,236]. It affects mostly children and it can compromise nutritional status resulting in stunted growth and impaired memory. Helminth infections is treated by combined drug regimen, improved sanitation and health education [235,236]. They are treated using anthelmintics agents however, some of these infections suffer from drug resistance. Some researchers have explored the potential of metal nanoparticles in treating these infections such as, silver and gold nanoparticles and metal-based oxide nanoparticles such as, zinc oxide and iron oxide (Table 6).

#### 4.3.1. Silver Nanoparticles

Silver nanoparticles have been combined with plant extracts resulting in good anthelmintic activity. Rashid et al., reported the anthelmintic activity of polyaniline-coated silver nanoparticles synthesized from *Momordica charantia* fruit extract [237]. The silver nanoparticles kill times of worms were 35.12 ± 0.5 and 59.3 ± 0.3 min for *plant* extract and the nanoparticles, respectively. Combining the nanoparticles and the plant extract resulted in enhanced anthelmintic activity against worm [237]. The positive charge on the silver ion can attract to the negatively charged cell membrane of microorganisms via electrostatic interaction. The plant extract contains phytochemicals that can attach with the free proteins in the gastrointestinal tract on the parasite cuticle resulting in death [237].

#### 4.3.2. Gold Nanoparticles

Apart from silver nanoparticles, gold nanoparticles are potential anthelmintic agents. Kar et al., evaluated gold nanoparticles anthelmintic activity [238]. Gold nanoparticles were prepared by treating gold chloride with mycelia-free culture filtrate of the phytopathogenic fungus. The diameter was between 6 and 18 nm. The gold nanoparticles affected the physiological functioning of the parasite causing paralysis and subsequent death. Alterations in the enzyme activity of the parasite after treatment with gold nanoparticles was significant, revealing the potential of gold nanoparticles [238].

#### 4.3.3. Metal Oxide Nanoparticles

Metal oxide nanoparticles such as, zinc oxide and iron oxide exhibit anthelminthic activity on helminth parasites. Zinc oxide nanoparticles anthelmintic effect on helminth parasite which infects Indian livestock was reported by Khan et al. [239]. Low concentrations of the nanoparticles at 80 μg/mL and 160 μg/mL produced oxidative stress by the production of ROS in the parasites. The flukes exhibited survival effort by increasing the activity of antioxidant enzymes to scavenge the ROS. The survival effort of the parasite was disrupted when the worms were treated with high concentration of 240 μg/mL of the nanoparticles. Saturation of antioxidant enzymes of the worm rendered the detoxification mechanism in *G*. *explanatum* ineffective. The elevated intracellular ROS level is believed to the permeability of the cell membrane, disrupt the electron transport system inhibiting ATP production and affect the contractile movement of the parasite [239]. Dorostkar et al., evaluated the antihelmintic activity of zinc oxide and iron oxide nanoparticles against *T. vitulorum* [240]. Iron oxide nanoparticles were more effective than zinc oxide nanoparticles which was attributed to the nature of the nanoparticles. Treatment with low dose of 0.004%, *w*/*v* of the both nanoparticles resulted in elevation of Superoxide Dismutase activity (SOD). At high concentration of 0.012%, *w*/*v* of the nanoparticles, a reduction of the SOD activity in the *T. vitulorum* was significant resulting from the saturation of the enzyme [240]. Oxidative stress caused by the nanoparticles at high concentration resulted in structural damage and overwhelms ATP production. The anthelmintic activity of the metal oxides nanoparticles is via induction of oxidative stress [240].

## 5. Conclusions and Future Trend

Infectious diseases are classified as bacterial, viral and parasitic and their treatment is hampered by drug resistance. Most of the drugs currently used for the treatment of infectious diseases suffer from drug toxicity and they are non-selective. Metal-based nanoparticles have been reported to have the potential to overcome these limitations. Metal-based nanoparticles exhibit good cellular interactions with biomolecules within the cell and on the cell surfaces. They can also be engineered by introducing selected biological moieties with specific binding activity to selected target cells thereby improving their therapeutic efficacy at the pathological site. Their excellent cellular interaction has been employed by several researchers to develop therapeutics for the treatment of several diseases, including infectious diseases. Most of the research reports on the metal-based nanoparticles for the treatment of infectious disease are based on preclinical evaluations. There is a serious need for these nanoparticles to reach clinical trials. The reported nanoparticles are characterized by enhanced cell uptake, good interaction with cell and some exhibit good selectivity when modified with selected functionalities. The antibacterial activity of metal-based nanoparticles is attributed to their ability to produce reactive oxygen species that damage the bacteria cell wall and their ability to bind to DNA or RNA, thereby hindering microbial replication process; disruption of bacterial enzyme and mitochondrial functions; and hindrance of electron transmembrane transport. The antibacterial activities of the metal-based nanoparticles were selective towards some strains of bacteria and were dependent on factors such as the size, concentration, shape, and method of preparation. The combination of the antibacterial agents with nanoparticles often results in good synergistic effects, except in the combination of ciprofloxacin with iron oxide nanoparticles that resulted in a poorer antibacterial activity of the nanoparticles suggesting that metal nanoparticles’ interaction with antibacterial agents may hinder the therapeutic efficacy of the antibacterial agents. More research is needed to understand the interaction of metal based nanoparticles with antibacterial drugs.

Metal-based nanoparticles have also been reported to have the potential to suppress co-viral infection, enhance the efficacy of anti-viral drugs, useful for prophylactic and therapeutic applications. They exhibit antiviral activity by interacting with the surface of virion thereby inhibiting the receptor binding sites on the virus and inhibit early stages of viral replication. However, there are very few reports, indicating that there is the need for more research in the design of metal nanoparticles for the treatment of viral infections. The potential of metal nanoparticles to treat parasitic infection have also been reported for infections such as malaria, leishmaniasis, and helminths. The antimalaria activity of metal nanoparticles has been reported to be good to moderate when compared to the available antimalarials. The antimalarial activity of the nanoparticles prepared from plant extracts is attributed to the presence of bioactive metabolites which act as anti-oxidative activity against oxidative stress induced in the host parasitized red blood cells by the malarial parasites. In the treatment of helminth infections, combining metal nanoparticles and plant extract resulted in enhanced anthelmintic activity. The positive charge on the metal ion was attracted to the negatively charged cell membrane of microorganisms via electrostatic interaction while the plant extract contains phytochemicals that attach with the free proteins in the gastrointestinal tract on the parasite cuticle resulting in parasitic death. The nanoparticles also altered the enzyme activity of the parasite resulting in parasitic death. High concentration of the nanoparticles was reported to be effective, resulting in an elevated level of intracellular ROS that permeates the cell membrane, disrupt the electron transport system and inhibit ATP production of the parasite.

Despite the aforementioned potential therapeutic efficacy of metal nanoparticles, some of the nanoparticles exhibited reduced biological activity which was attributed their design, nature of the metal and poor selectivity towards the target cells. These limitations were overcome in cases where the metal compounds were incorporated into selected drug delivery systems. Reports on the application of metal-based nanoparticles for the treatment of parasitic infection is low when compared to other infectious diseases, indicating there is an urgent need to develop metal-based nanoparticles that are affordable with high therapeutic outcomes. There is also a need to study the toxicological properties and pharmacokinetics of metal-based compounds. Metal-based nanoparticles have the potential to overcome drug resistance which is common with most organic molecules. There is no doubt that metal-based nanoparticles are promising future therapeutics for the treatment of infectious diseases.

## Figures and Tables

**Table 1:** Silver nanoparticles with antibacterial activity.

Metal Nanoparticles	Therapeutic Outcome	References
Silver nanoparticles	Effective against *Escherichia coli* and *Staphylococcus aureus.*	[24,25,26,27,28,29,30]
Sphere-shaped, and triangle shaped silver nanoparticles	The antibacterial activity of the nanoparticles against *P. aeruginosa* bacteria was enhanced for spherically shaped nanoparticles. The triangle-shaped silver nanoparticles exhibited enhanced antibacterial effect which is attributed to high-atom-density facets and interaction of the facets with the surface of the bacteria.	[31,32,33,34]
Rod-shaped silver nanoparticles	Triangular shaped nanoparticles exhibited high antibacterial activity against *Escherichia coli* than the spherical and rod shaped silver nanoparticles.	[32]
Hexagonal and nanoplates silver nanoparticles	Hexagonal-shaped silver nanoparticles were effective against *S. aureus* and *E. coli* when compared to the nanorod- and nanoplate-shaped silver nanoparticles.	[35,36]
Nanocube and nanowire-shaped silver nanoparticles	Nanocube-shaped silver nanoparticles exhibited the highest antibacterial activity because of their surface area, effective contact area, and facet reactivity.	[37]
Silver nanoparticles	Inhibited the growth of *Mycobacterium tuberculosis*	[38,39,40]
Silver nanoparticles	Effective against bacteria causing sexually transmitted disease e.g., *Chlamydia trachomatis*.	[41,42]
Silver nanoparticles in urinary catheter	Effective against bacteria that are responsible for urinary tract infections.	[43,44,45,46,47,48]
silver nanoparticles combined with polymixin B and rifampicin	Good synergistic effects in the treatment of *Acinetobacter baumannii* infection.	[49]
Silver nanoparticles combination with amoxicillin	Good synergistic effects against *Escherichia coli* resulting from chelation between active groups such as hydroxy and amido groups on amoxicillin with the nanosilver.	[50]
Silver nanoparticles combination with β-lactam; quinolone; aminoglycoside and polykeptide	Effective against drug-resistant bacteria *Salmonella typhimurium.* β-lactam class of antibiotics did not show synergistic effects because of its inability to form a complex with the nanoparticles.	[51]
Silver nanoparticles combination with gentamicin and penicillin	Excellent antibacterial effects against animal bacterial infections, *Actinobacillus pleuropneumoniae*, *A. pleuropneumoniae* and *Pasteurella multocida.*	[52]
Silver nanoparticles combination with visible blue light and either amoxicillin, azithromycin, clarithromycin, linezolid or vancomycin	Good synergistic antibacterial effects against methicillin-resistant *Staphylococcus aureus*.	[53]
Silver nanoparticles combined with either cefazolin, mupirocin, gentamycin, neomycin, tetracycline or vancomycin	Combination of nanoparticles with antibiotics was effective against *Staphylococcus aureus*, *Pseudomonas aeruginosa* and *Escherichia coli.*	[54]
Conjugation of cephalexin onto silver nanoparticles	Effective against *E. coli* and *S. aureus* by binding to the cell wall resulting in the destruction of the cell outer membrane.	[55]
Silver nanoparticles prepared using plants extracts	Stable nanoparticles with good antibacterial activity.	[56,57,58,59,60,61,62,63,64,65,66]
Silver nanoparticles prepared by biological methods using virus, bacteria and fungi	Good antibacterial activity.	[67,68,69,70,71,72,73]

**Table 2:** Iron oxide and copper oxide nanoparticles with antibacterial activity.

Metal Nanoparticles	Therapeutic Outcome	References
Iron oxide nanoparticles	Good antibacterial activity on *E. coli*, *P. vulgaris*. *Staphylococcus aureus*	[74,75,76,77,78]
Iron oxide nanoparticles combination with erythromycin	Good synergistic antibacterial effects against *S. pneumonia*	[79]
Iron oxide nanoparticles	Inhibition of growth of *Staphylococcus aureus*, *Escherichia coli*, *Pseudomonas aeruginosa* and *Serratia marcescens*	[80,81]
Iron oxide nanoparticles coated with chitosan biomolecules	Good inhibition of growth of *Bacillus subtilis* and *Escherichia coli* bacteria	[82]
Iron oxide nanoparticles using *Punica granatum* peel extract	Strong antibacterial activity against *Pseudomonas aeruginosa*	[83]
Iron oxide nanoparticles	Good antibacterial effects against *Staphylococcus aureus* which is dependent on concentration	[85]
Chitosan coated iron nanoparticles	Inhibited the growth of *Escherichia coli* and *Salmonella enteritidis*	[86]
Iron oxide nanoparticles combined with ciprofloxacin	Poor antibacterial activity	[87]
*Iron oxide nanoparticles prepared using* leaf extract	Good antibacterial with varied shapes	[88,89,90,91,92,93,94,95,96,97]
*Copper oxide nanoparticles*	Very sensitive to *E. coli* and *E. faecalis* and less selective to *K. pneumonia*	[98]
copper oxide nanorods and multi-armed nanoparticles	Multi-armed nanoparticles exhibited higher antibacterial activity against *E. coli* than the nanorods	[99]
Copper oxide nanoparticles	The antibacterial activity of copper oxides is attributed to lipid peroxidation, generation of reactive oxygen species, protein oxidation and DNA degradation in bacteria cells	[100]
Copper oxide nanoparticles	antibacterial of the nanoparticles was dependent on the particle sizes	[101,102]
Copper oxide nanoparticles	The nanoparticles exhibited spherical shapes with high antibacterial activities against *Bacillus subtilis* and *Salmonella choleraesuis*	[103]
Copper oxide nanoparticles	The antibacterial activity of the nanoparticles was effective against *K. pneumoniae, S. typhimurium,* and *E. aerogenes*	[104]
Copper oxide nanoparticles	Effective against different strains of *Staphylococcus aureus*	[105]
Copper oxide nanoparticles	Good antibacterial activity of copper oxide nanoparticles against Gram-positive (*B. subtilis* and *S. aureus*) and Gram-negative (*E. coli* and *P. aeruginosa*) bacteria	[106]
Copper oxide nanoparticles	Good antibacterial activity against *Escherichia coli and Pseudomonas aeruginosa*	[107]
Copper oxide nanoparticles	Effective against *Escherichia coli* and *Lactobacillus brevis*	[108,109,110,111,112,113,114]

**Table 3:** Zinc oxide and aluminium oxide nanoparticles antibacterial activity.

Metal Nanoparticles	Therapeutic Outcome	References
Zinc oxide nanoparticles	Good antibacterial activity against *Klebsiella pneumonia* that causes respiratory infection	[115]
Zinc oxide nanoparticles	The inhibition effect on the growth of *B*. *subtilis* was dependent on the concentration of the nanoparticles	[116,117]
Zinc oxide nanoparticles	The antibacterial effect against clinical isolate of *Staphylococcus aureus* was excellent	[118]
Zinc oxide nanoparticles	Effective against *Campylobacter jejuni*	[119]
Zinc oxide nanoparticles	Good antibacterial activity by ROS mediated membrane lipid oxidation of *Escherichia coli*, *S. aureus*, *P. aeruginosa* and *V. anguillarum*	[120,121,122,123]
Zinc oxide nanoparticles	Effective against *E. coli*	[124,125,126]
Zinc oxide nanoparticles	Effective against Gram-positive bacteria. The antibacterial effect was high on *B. subtilis* cells when compared to *S. aureus*	[127]
Zinc oxide nanoparticles coated with gentamicin	The antibacterial effects against *Escherichia coli*, *Pseudomonas aeruginosa*, *Staphylococcus aureus*, *Bacillus cereus* and *Listeria monocytogenes* was significant	[128]
Zinc oxide nanoparticles using aqueous extracts of *P. crispum*	Excellent antibacterial activity	[129]
Zinc oxide nanoparticles prepared from plants extract	Enhanced antibacterial activity	[130,131]
Aluminium oxide nanoparticles	*Nanoparticles penetrated Candida* cells disrupting the morphological and physiological activity of the cells.	[132,133]
aluminium oxide nanoparticles prepared from leaf extracts of lemongrass	Good antibacterial, activity against clinical isolates of *P. aeruginosa* was significant	[134]
Aluminium oxide nanoparticles	Effective against gram-positive and gram-negative bacteria	[135,136]

**Table 4:** Gold, titanium dioxide and gallium nanoparticles with antibacterial activity.

Metal Nanoparticles	Therapeutic Outcome	References
Gold nanoparticles	*Effective against bacterial infection in animal*, *Corynebacterium pseudotuberculosis*	[137]
Gold nanoparticles	Effective against *E. Coli*	[138,139,140,141,142]
Gold nanoparticles	The nanoparticles were active against Gram-negative, Gram-positive uropathogens and multi-drug resistant pathogens	[139]
Gold nanoparticles	Active against enteric bacteria e.g., *Escherichia coli, Staphylococcus aureus*, *Bacillus subtilis and Klebsiella pneumonia*	[140]
Gold nanoparticles	Effective against *E. coli*, *S. typhimurium DT104*, and *S. aureus*	[141]
Gold nanoparticles	Inhibited growth of *Salmonella typhi*	[142]
Gold nanoparticles	Effective against *Staphylococcus aureus* and *Escherichia coli*	[143]
Gold nanoparticles combined with gentamicin	*Effective against Escherichia coli*	[144,145]
Gold nanoparticles capped with cefaclor	Potent antimicrobial activity against both Gram-positive (*Staphylococcus aureus*) and Gram-negative (*Escherichia coli*) bacteria	[145]
Gold nanoparticles	Potent antibacterial effect against multidrug-resistant Gram-negative bacteria	[146]
Gold nanoparticles prepared using banana peel extract	Good antibacterial activity	[147]
Gold nanoparticles combined with ofloxacin	Superior bactericidal property	[148]
Gold nanoparticles prepared using Stoechospermum marginatum	Enhanced antibacterial activity	[149]
Gold nanoparticle prepared from A. comosus extract	Useful purification processes for inhibiting the growth of bacteria	[150]
Gold nanoparticles prepared using aqueous leaves extract of *Moringa oleifera*	Effective against *Staphylococcus aureus*, *Candida tropicalis*, *Candida krusei*, *Klebsiella pneumonia*	[151]
Gold nanoparticles	Active against *Escherichia coli*	[152]
Gold nanoparticles combined with gentamicin	Good antibacterial activity	[153]
light-absorbing gold nanoparticles conjugated with specific antibodies	selective killing of the Gram-positive bacterium *Staphylococcus aureus*	[154,155,156,157]
Gold nanoparticles combined with vancomycin	Selective binding to the cell of Gram-positive bacteria, Gram-negative bacteria and antibiotic-resistant bacteria	[156,157]
Gold nanoparticles combined with ampicillin	Effective against *E. coli*, *Micrococcus luteus* and *Staphylococcus aureus*	[158]
Gold nanoparticles combined with streptomycin	Effective against *E. coli*, *Micrococcus luteus* and *Staphylococcus aureus*	[158]
Gold nanoparticles combined with kanamycin	Effective against *E. coli*, *Micrococcus luteus* and *Staphylococcus aureus*	[158]
Gold nanoparticles combined with levofloxacin	Inhibited growth of *S. aureus* and *E. coli*	[159]
Titanium dioxide nanoparticles	Effective against biofilm producing methicillin-resistant *S. aureus*	[160]
Titanium dioxide nanoparticles	Inhibited growth of bacteria that causes dental plaques	[161]
Titanium dioxide nanoparticles	Effective against *Streptococcus mutans*	[163]
Titanium dioxide nanoparticles	Effective against *E. coli*	[164,165,166,167,168]
Gallium nanoparticles	Inhibited the growth of mycobacteria	[169]
Gallium nanoparticles	Good antibacterial properties against *Pseudomonas aeruginosa*	[170]
Gallium nanoparticles	*Inhibited growth of Mycobacterium tuberculosis* significantly	[171]
Gallium nanoparticles	*Disrupted F. Tularensis* Fe metabolism	[172]

**Table 5:** Metal-based nanoparticles with antiviral activity.

Nanoparticles	Infection	Therapeutic Outcome	References
Silver nanoparticles	HIV	Inhibition of CD4-dependent virion binding, fusion, and infectivity	[173,174,175,176,177]
Gallium nanoparticles	HIV	Suppressed co-infection of HIV and tuberculosis. Inhibition of viral protease	[178,179,180]
Gold nanoparticles combined with Azidothymidine	HIV	Inhibition of early stages of viral replication	[181,182]
Gold nanoparticles conjugated with raltegravir	HIV	Good anti-HIV activity	[183]
Gold nanoparticles	HIV	Inhibition of viral entry	[184]
Carbohydrate-coated gold nanoparticles conjugated with abacavir and lamivudine	HIV	The nanoparticles inhibited HIV viral replication	[185,186]
Peptide triazoles conjugated onto gold nanoparticle	HIV	Potent antiviral effects against HIV-1	[187]
Tin nanoparticles	Herpes	Trapped HSV-1 before entry into the host cell	[188,189,190]
Silver nanoparticles	Herpes	Virus replication was inhibited	[191]
Tannic acid modified with silver nanoparticles	Herpes	reduced HSV-2 infection	[192]
Silver nanoparticles	Herpes	Inhibition of viral entry into the cell and prevention of subsequent infection	[193]
Polyurethane condom coated with silver nanoparticles	Herpes	Inhibition of HSV-1 and 2 infections	[194,195]
Zinc oxide	Herpes	Prevented viral entry and infection	[196,197]
Gold nanoparticle	Herpes	Inhibited viral attachment and penetration into the cells thereby preventing infections	[198,199]
Silver nanoparticles	Hepatitis	Interaction with the HBV viral particles resulting in the inhibition of the production of HBV RNA and extracellular virions	[200,201]
Iron oxide nanoparticles	Hepatitis	Induced the knockdown of hepatitis C virus gene, NS3. HCV NS3 gene encodes helicase and protease which are useful for viral replication	[202]
Cuprous nanoparticle	Hepatitis	Inhibited the entry of virus which included genotypes such as, 1a, 1b, and 2a thereby hindering viral replication	[203]
Gold nanoparticle loaded with interferon α	Hepatitis	Targeted delivery of interferon α	[204,205]
Gold and silver nanoparticles	Influenza	Effective against influenza A virus	[206,207]
Silver nanoparticles	Influenza	Effective against influenza viruses resulting in damage to their morphological structure. Inhibiting the host receptor binding sites of the virus	[208,209,210,211,212,213]

**Table 6:** Metal-based nanoparticles for the treatment of parasitic infections.

Nanoparticles	Infection	Therapeutic Outcome	References
Silver nanoparticles	Malaria	Inhibition of the growth of *P. falciparum* in vivo and in vitro	[216,217,218,219]
Metal oxide nanoparticles (Fe_3_O_4_, MgO, ZrO_2_, Al_2_O_3_ and CeO_2_)	Malaria	Good to moderate antiplasmodial activity against *P. falciparum*	[220]
Gold nanoparticles	Malaria	Moderate delayed parasitemia rise in vivo, moderate antiplasmodial activity against *P. falciparum*	[221,222]
Silver nanoparticles	Leishmaniasis	Inhibition of proliferation and metabolic activity of promastigotes. Good antileishmanial activity in vitro and in vivo	[223,224,225,226,227,228,229]
Kaempferol-stabilized gold nanoparticles	Leishmaniasis	Effective against both wild and drug resistant strains	[230,231]
Metal-oxide nanoparticles (titanium dioxide nanoparticles, zinc oxide nanoparticles and magnesium oxide nanoparticles)	Leishmaniasis	Enhanced cytotoxic effects on promastigotes of *L. major* via induction of apoptosis	[232,233]
Silver nanoparticles	Helminth infections	Enhanced anthelmintic activity against worm	[234,235,236,237]
Gold nanoparticles	Helminth infections	Affected the physiological functioning of the parasite causing paralysis and subsequent death	[238]
Zinc oxide nanoparticles	Helminth infection	Disruption of the electron transport system inhibiting ATP production and the contractile movement of the parasite	[239]
Zinc oxide and iron oxide nanoparticles	Helminth infection	The anthelmintic activity of the metal oxides nanoparticles was via induction of oxidative stress	[240]
